# Supplementary material for: Factors influencing dignity impairment in elderly patients with incontinence-associated dermatitis: A lasso and logistic regression approach
Source: PLoS One. 2025 Apr 10;20(4):e0320319. doi: 10.1371/journal.pone.0320319 (PMC11984707; doi:10.1371/journal.pone.0320319)
Supplement: S1 Fig — (DOCX) [file pone.0320319.s001.docx]

**
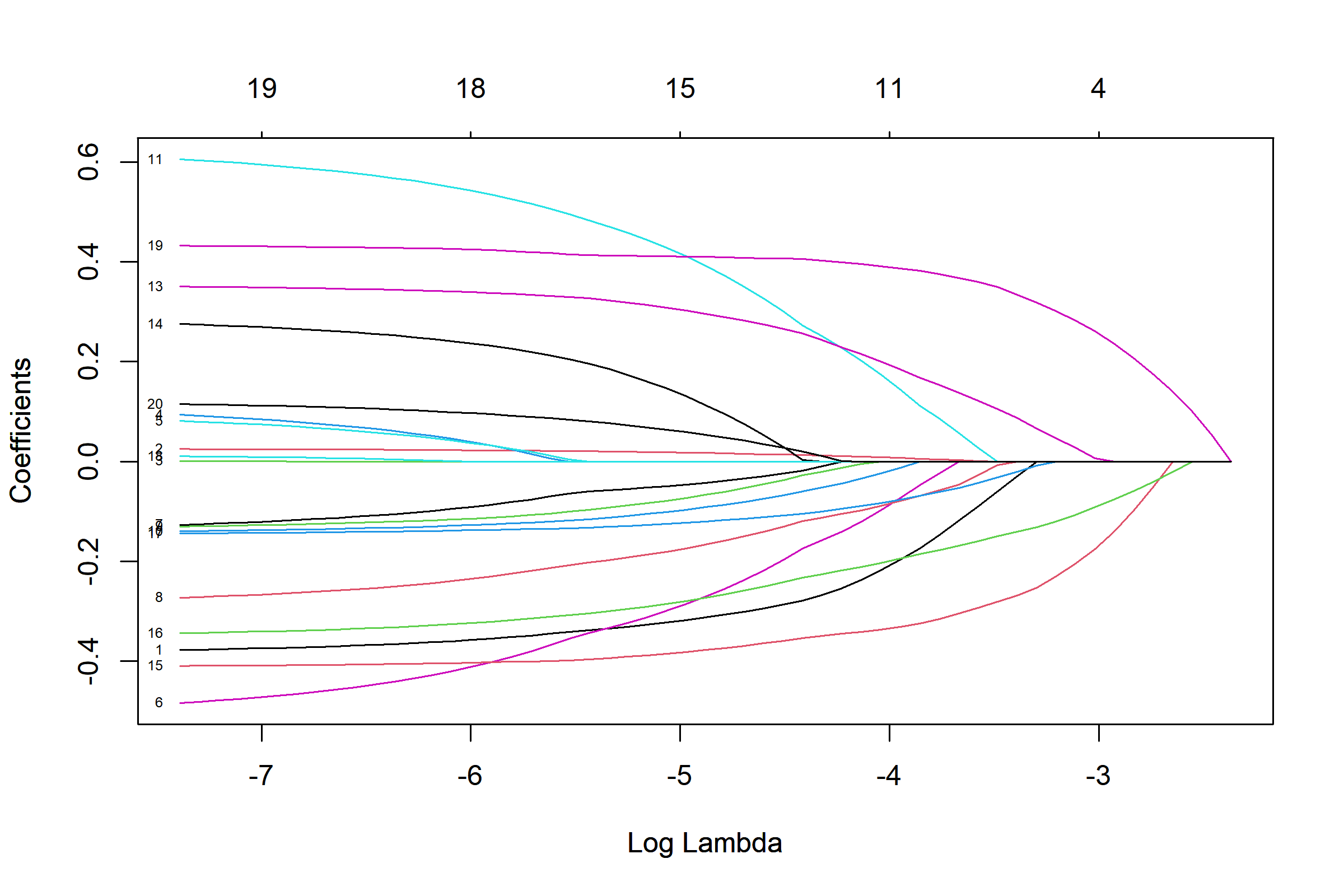
**

# Fig 1 .Lasso Coefficient Path for Variables in Assessing Dignity Impairment Symptoms in Elderly Patients with Incontinence-Associated Dermatitis
