# Supplementary material for: Factors influencing dignity impairment in elderly patients with incontinence-associated dermatitis: A lasso and logistic regression approach
Source: PLoS One. 2025 Apr 10;20(4):e0320319. doi: 10.1371/journal.pone.0320319 (PMC11984707; doi:10.1371/journal.pone.0320319)
Supplement: S1 Table — (DOCX) [file pone.0320319.s002.docx]

# Table 1. Variables Selected by LASSO Analysis for Assessing Dignity Impairment Symptoms in Elderly Patients with Incontinence-Associated Dermatitis

| Variable | Group | Sample Size with Dignity Loss  （n=131） | | Sample Size without Dignity Loss  （n=241） | | LASSO Coefficient | | Included in Model |
| --- | --- | --- | --- | --- | --- | --- | --- | --- |
| Gender No(%) | Male | | 66(50.38) | | 98(40.66) | | -0.218 | Yes |
|  | Female | | 65(49.62) | | 143(59.34) | |  |  |
| Age  M（Q_25,_Q_75_） |  | | 71(68,78) | | 70(67,77) | | 0.008 | Yes |
| Body mass index  M（Q_25,_Q_75_） |  | | 24.60(22.90,25.30) | | 24.50(22.90,25.90) | | 0.000 | No |
| Ethnicity No(%) | Ethnic han | | 123(93.89) | | 222(92.11) | | 0.000 | No |
|  | Other ethnic groups | | 8(6.11) | | 19(7.89) | |  |  |
| Educational Level No(%) | Primary and below | | 56(42.74) | | 95(39.41) | | 0.000 | No |
|  | Junior and senior high schools | | 45(34.35) | | 81(33.60) | |  |  |
|  | College and above | | 30(22.91) | | 65(26.99) | |  |  |
| Place of Residence No(%) | Rural | | 85(64.88) | | 151(62.65) | | -0.098 | Yes |
|  | Urban | | 46(35.12) | | 90(37.35) | |  |  |
| Marital Status No(%) | Married | | 99(75.57) | | 184(76.34) | | 0.000 | No |
|  | Non-married | | 32(24.43) | | 57(23.66) | |  |  |
| Personality Traits No(%) | introverted | | 96(73.28) | | 141(58.50) | | -0.088 |  |
|  | outgoing | | 35(26.72) | | 100(41.50) | |  |  |
| Religious Beliefs No(%) | None | | 54(41.22) | | 162(67.21) | | 0.000 | No |
|  | Yes | | 77(58.78) | | 79(32.79) | |  |  |
| Primary Occupation No(%) | Staff of public institutions | | 38(29.00) | 67(27.80) | | | -0.024 | Yes |
|  | Agriculture | | 55(41.98) | 52(21.57) | | |  |  |
|  | Financial business | | 25(19.08) | 72(29.87) | | |  |  |
|  | Others | | 13(9.94) | 50(20.76) | | |  |  |
| Employment Status No(%) | Employed | | 107(76.33) | 142(58.92) | | | 0.174 | Yes |
|  | Retired | | 24(23.67) | 99(41.08) | | |  |  |
| Payment Method for Medical Expenses No(%) | Urban medical insurance | | 84(64.12) | 148(61.41) | | | 0.000 | No |
|  | Employee medical insurance | | 29(22.13) | 63(26.14) | | |  |  |
|  | Others | | 18(13.75) | 30(12.45) | | |  |  |
| Primary Caregiver No(%) | Spouse | | 51(38.93) | 123(51.03) | | | 0.200 | Yes |
|  | Children | | 60(45.80) | 89(36.92) | | |  |  |
|  | Others | | 20(15.27) | 29(12.95) | | |  |  |
| Monthly Personal Income No(%) | ≤3000 | | 83(63.35) | 122(50.62) | | | 0.000 | No |
|  | ＞3000 | | 48(36.65) | 119(49.38) | | |  |  |
| Living Situation No(%) | Live alone | | 83(63.35) | 59(24.48) | | | -0.336 | Yes |
|  | Non-solitary | | 48(36.65) | 182(75.52) | | |  |  |
| Self-Reported Family Harmony No(%) | Not harmonious | | 29(22.13) | 42(17.42) | | | -0.203 | Yes |
|  | Relatively harmonious | | 49(37.40) | 70(29.04) | | |  |  |
|  | Harmonious | | 37(28.24) | 56(23.23) | | |  |  |
|  | Very harmonious | | 16(12.23) | 73(30.31) | | |  |  |
| Primary Disease No(%) | Digestive system | | 29(22.13) | 79(32.78) | | | -0.082 | Yes |
|  | Respiratory system | | 40(30.53) | 68(28.21) | | |  |  |
|  | Nervous system | | 45(34.35) | 76(31.53) | | |  |  |
|  | Other systemic | | 17(12.99) | 18(7.48) | | |  |  |
| Duration of IAD No(%) | Under a week | | 64(48.85) | 147(60.99) | | | 0.000 | No |
|  | One to two weeks | | 45(34.35) | 55(22.82) | | |  |  |
|  | More than two weeks | | 22(16.80) | 39(16.19) | | |  |  |
| Grading of IAD No(%) | Level o | | 41(31.29) | 133(55.18) | | | 0.391 | Yes |
|  | Level 1 | | 70(53.43) | 77(31.95) | | |  |  |
|  | Level 2 | | 20(15.28) | 31(12.87) | | |  |  |
| Frequency of Daily Care or Cleaning No(%) | Less than 3 times | | 40(30.53) | 68(28.21) | | | 0.000 | No |
|  | Between 3 and 5 times | | 52(39.69) | 98(40.66) | | |  |  |
|  | More than 5 times | | 39(29.78) | 75(31.13) | | |  |  |
