# Supplementary material for: Factors influencing dignity impairment in elderly patients with incontinence-associated dermatitis: A lasso and logistic regression approach
Source: PLoS One. 2025 Apr 10;20(4):e0320319. doi: 10.1371/journal.pone.0320319 (PMC11984707; doi:10.1371/journal.pone.0320319)
Supplement: S2 Table — (DOCX) [file pone.0320319.s003.docx]

# Table 2. Assignment of Independent Variable Values

| Variant | Assignment method |  |
| --- | --- | --- |
| Gender | Male=1,Female=2  Original value input  Rural=1,Urban=2  Staff of public institutions=(0,0,0),Agriculture=(1,0,0),  Financial business=(0,1,0),Others=(0,0,1) | |
| Age |  |  |
| Place of Residence |  |  |
| Primary Occupation |  |  |
|  |  |  |
| Employment Status | Employed=1,Retired=2 | |
| Primary Caregiver | Spouse=1,Children=2,Others=3 | |
| Living Situation | Live alone=1,Non-solitary=2 | |
| Self-Reported Family Harmony | Not harmonious=1,Relatively harmonious=2,Harmonious  =3,Very harmonious=4 | |
| Primary Disease | Digestive system=(0,0,0),Respiratory system=(1,0,0),Nervous system=(0,1,0),Other systemic=(0,0,1) | |
| Grading of IAD | Level o=1,Level 1=2,Level 2=3 | |
