# Supplementary material for: Factors influencing dignity impairment in elderly patients with incontinence-associated dermatitis: A lasso and logistic regression approach
Source: PLoS One. 2025 Apr 10;20(4):e0320319. doi: 10.1371/journal.pone.0320319 (PMC11984707; doi:10.1371/journal.pone.0320319)
Supplement: S3 Table — (DOCX) [file pone.0320319.s004.docx]

# Table 3. Multifactorial Analysis Results of Dignity Impairment Symptoms in Elderly Patients with Incontinence-Associated Dermatitis

| Variable |  | B | SE B | P | OR | 95%CI |
| --- | --- | --- | --- | --- | --- | --- |
| Gender |  | -0.718 | 0.256 | 0.005 | 0.488 | 0.295-0.807 |
| Employment Status |  | 1.431 | 0.320 | ＜0.001 | 4.183 | 2.233-7.837 |
| Primary Caregiver |  | 0.372 | 0.187 | 0.047 | 1.451 | 1.005-2.095 |
| Self-Reported Family Harmony |  | -0.353 | 0.125 | 0.005 | 0.703 | 0.550-0.898 |
| Primary Disease | Digestive system* |  |  |  |  |  |
|  | Respiratory system | 1.620 | 0.453 | ＜0.001 | 5.053 | 2.079-12.279 |
|  | Nervous system | 0.897 | 0.363 | 0.013 | 2.452 | 1.206-4.985 |

Note: Hosmer-Lemeshow Goodness-of-fit Test:χ^2^=11.598,*P*=0.170
