# Supplementary material for: Factors influencing dignity impairment in elderly patients with incontinence-associated dermatitis: A lasso and logistic regression approach
Source: PLoS One. 2025 Apr 10;20(4):e0320319. doi: 10.1371/journal.pone.0320319 (PMC11984707; doi:10.1371/journal.pone.0320319)
Supplement: Attachment — Submitted filename: Response to Editor and reviewers.docx [file pone.0320319.s006.docx]

Manuscript Title: Univariate Screening and Multivariate Analysis of Factors Influencing Dignity Impairment in Elderly Patients with Incontinence-Associated Dermatitis: A Lasso and Logistic Regression Approach

Dear Editor:

Thank you for your letter and for the reviewers’ comments concerning our manuscript entitled“Univariate Screening and Multivariate Analysis of Factors Influencing Dignity Impairment in Elderly Patients with Incontinence-Associated Dermatitis: A Lasso and Logistic Regression Approach”.Those comments are all valuable and very helpful for revising and improving our paper, as well as the important guiding significance to our research. We have studied comments carefully and have made correction with the hope of approval. Revised portion are marked in red in the paper.

The main corrections in the paper and the responds to the editor and reviewer’s comments are as following:

**Responds to the editor’s comments:**

1 It needs grammar edition (capitalization,…)

Title should be modified as - Factors Influencing Dignity Impairment in Elderly Patients with Incontinence-Associated Dermatitis: A Lasso and Logistic Regression Approach

Limited keywords- add more words

Response:Dear Editor,Thank you for your constructive suggestions regarding the title and keywords of our manuscript. In response, we have revised the title to "Factors Influencing Dignity Impairment in Elderly Patients with Incontinence-Associated Dermatitis: A Lasso and Logistic Regression Approach", which better reflects the focus and methodology of our study. Additionally, we have expanded the keywords to include "Incontinence-Associated Dermatitis,Elderly Patients,Dignity Impairment,Chronic Diseases,LASSO Regression,Logistic Regression", ensuring improved discoverability and alignment with the scope of the journal. All changes have been marked in red within the manuscript for clarity. We sincerely appreciate your thoughtful consideration and believe these revisions enhance the manuscript’s clarity and relevance.

2 Introduction

In line 35 a statement: This condition can affect skin areas beyond the perineum. Have no reference

Statement in line 45-47 - Current research both domestically and internationally mainly focuses on the 46 prevention and treatment of IAD symptoms, with limited studies addressing 47 psychological aspects, especially in the domain of dignity. Cite reference

Response:Dear Editor,Thank you for your valuable feedback. Based on your suggestions, we have inserted the appropriate references in two places.We sincerely appreciate your insightful comments, which have helped improve the quality of the manuscript.

3 General: the introduction part should be clearly stated and written with sufficient information

Problem statement of Dignity Impairment

Potential significance of your study

Use appropriate reference

Response:Dear Editor,Thank you for your constructive suggestions regarding the introduction section of our manuscript. In response to your feedback, we have made the following revisions: We have enhanced the problem statement of dignity impairment by providing more detailed information on its definition, significance, and impact on patients, supported by relevant references. Additionally, we have clearly outlined the potential significance of our study, emphasizing its importance in improving clinical care and addressing a gap in the existing literature, with appropriate citations. All changes have been marked in red within the manuscript for clarity. We sincerely appreciate your thoughtful consideration and believe these revisions enhance the manuscript’s clarity and depth.

4 Materials and Method

In line 85: Exclusion criteria: you stated that history of depression within the past three months; diagnosis 86 of psychiatric disorders; presence of other severe, incurable chronic diseases; 87 participation in concurrent psychological intervention studies. Why 3 months? Do you mean that psychiatric disease onset within three months does not affect the results?

Response:Dear Editor,Thank you for your insightful question regarding the exclusion criteria in our study. The rationale for setting the "history of depression within the past three months" exclusion criterion is based on several considerations.

First, depression is a complex and dynamic condition that can significantly affect an individual’s psychological state. To ensure participants’ mental health is relatively stable and not influenced by recent depressive episodes, we chose a three-month timeframe. This period is generally considered sufficient for the effects of an acute depressive episode to subside, reducing potential confounding effects on the study outcomes.Second, we also excluded participants with a diagnosis of psychiatric disorders and other severe, incurable chronic diseases to maintain sample homogeneity. These conditions could introduce variables that may interfere with the interpretation of our results.We believe that this exclusion criterion is necessary to ensure the internal validity of our study while maintaining a representative sample.We hope this explanation clarifies the reasoning behind our exclusion criterion. We are happy to provide further details if needed.

5 Line 88: This study has been 88 approved by the hospital’s ethics committee: remove from this part

Response:Dear Editor,Thank you for your valuable feedback. In response to your suggestion, we have removed the statement regarding the approval by the hospital’s ethics committee from line 88 as per your recommendation.We appreciate your guidance, which has helped to further refine the manuscript.

6 Discussion

Avoid subtitle in the discussion part

The discussion part is lacking, it needs more scientific justification

Response:Dear Editor,Thank you for your valuable feedback. In response to your suggestion to remove the subtitles in the discussion and to enhance the scientific justification, we have carefully revised this section. We have removed the subtitles as per your recommendation to improve the coherence and overall structure of the discussion. Additionally, we have strengthened the scientific justification by providing a deeper analysis of the study results and integrating relevant literature to support our findings. We further elaborated on how factors such as incontinence-associated dermatitis, self-care ability, social support, and caregiver identity contribute to dignity impairment in elderly patients. These revisions aim to provide a more comprehensive and scientifically grounded discussion, linking our findings with existing knowledge and offering practical implications for clinical care. We greatly appreciate your insightful suggestions, which have been instrumental in refining our manuscript. Should you have any further recommendations, we would be happy to make additional adjustments.

**Responds to the reviewer’s comments:**

**Reviewer 1:**

1.The cross-sectional design limits causal inferences about risk factors.There's limited information on the validity of the dignity assessment scale used.Some potential confounding factors (e.g. IAD severity, comorbidities) appear not to have been controlled for.Addressing these issues would strengthen the conclusions.

Response:Dear reviewer,Thank you for your valuable feedback and thoughtful consideration of our study. We fully understand your concern regarding the limitations of causal inferences due to the cross-sectional design. Indeed, cross-sectional studies provide data at a single time point and cannot establish the temporal sequence between variables, making it difficult to draw causal conclusions. However, our study still provides valuable correlational insights. Based on your guidance, we plan to expand our research further with a longitudinal design to clarify the causal relationships in future studies. Additionally, to address this limitation more clearly, we have added a detailed discussion of the cross-sectional study's constraints in the discussion section, along with suggestions for future research.

We also greatly appreciate your feedback regarding the dignity assessment scale used in our study. The Dignity Inventory, developed by Canadian scholar Chochinov, has demonstrated strong reliability and validity. Specifically, it has a content validity index of 0.885 and a Cronbach's α coefficient of 0.924, indicating its excellent usability in assessing dignity impairment symptoms. To enhance clarity, we have supplemented the assessment criteria for dignity impairment symptoms within the scale. Furthermore, we have included relevant references to support the validity and reliability of the scale.

Regarding potential confounding factors such as the severity of incontinence-associated dermatitis (IAD) and comorbidities, we appreciate your suggestion to address these more explicitly. The severity of IAD was indeed included in our study and assessed through IAD grading in the general information analysis section. To ensure transparency, we have added the reference standard for IAD grading in the manuscript. While we did not explicitly list comorbidities in the analysis, we would like to point out that these factors are inherently considered within the IAD grading system, which reflects overall health status. This approach allows for a comprehensive evaluation of the patients' conditions without the need for separate listing of comorbidities.

We hope these clarifications address your concerns and enhance the overall clarity of our study. Once again, thank you for your valuable feedback, which has greatly contributed to refining our manuscript.All modifications have been highlighted in red within the manuscript.

2.Statistical analysis

The statistical analysis appears appropriate and rigorous. It is appropriate to use Lasso regression followed by logistic regression to identify risk factors. The sample size calculation and reporting of odds ratios with confidence intervals are appropriate. However, more details on the specific statistical procedures would be beneficial.

Response:Dear Reviewer,Thank you for your insightful comments and for recognizing the rigor of our statistical analysis. We appreciate your suggestion to provide more detailed information on the specific statistical procedures used in our study.To address this, we have expanded our explanation of the statistical methods employed. Specifically, we have clarified the steps taken during the LASSO regression analysis, the process of variable selection, the diagnostics for collinearity, and the assessment of model fit using the Hosmer-Lemeshow test. Additionally, we have outlined how the final selected variables were subsequently analyzed through binary logistic regression.All the changes we made to the manuscript to improve clarity are highlighted in red font for easy reference.We hope this additional information strengthens the transparency and clarity of our statistical analysis. Thank you once again for your valuable feedback.

3 Additional comments:

Provide more details on how IAD was diagnosed and graded.

Clarify the validity and reliability of the dignity assessment scale used.

Discuss potential confounding factors not accounted for in the analysis.

Response:Dear Reviewer,Thank you for your constructive comments. We appreciate your suggestions, which allow us to provide more comprehensive and clear information in our manuscript. We have addressed your points as follows:

（1）Detailed Diagnosis and Grading of IAD: In response to your comment regarding the diagnosis and grading of IAD, we have added more detailed information on the diagnostic process and grading criteria in the manuscript. The diagnosis of IAD was based on clinical examination by trained healthcare professionals, primarily focusing on skin changes such as erythema, skin breakdown, and associated complications. We adopted the grading system outlined in the 2015 International Expert Consensus on Incontinence-Associated Dermatitis, which classifies IAD as Level 0 (intact skin with no erythema), Level 1 (intact skin with erythema, with or without edema), and Level 2 (erythema with skin breakdown, edema, vesicles, erosions, or infection). This grading system has been clearly stated in our manuscript.

（2）Validity and Reliability of the Dignity Assessment Scale: Regarding the validity and reliability of the dignity assessment tool used in our study, we have clarified the reliability and validity of the Dignity Inventory. The Dignity Inventory, developed by Canadian scholar Chochinov, has demonstrated strong validity and reliability. Specifically, the content validity index of the scale is 0.885, and its Cronbach's α coefficient is 0.924, indicating excellent internal consistency. These results provide strong evidence for the reliability and validity of the scale in our study. In addition, we have referenced relevant literature to further support the scale's validity and reliability.

（3）Further Analysis of Potential Confounding Factors: Following your guidance, we have expanded the discussion section to include a more thorough analysis of potential confounding factors that could affect the results. All modifications are marked in red font.

We hope that these revisions adequately address your concerns and enhance the clarity and rigor of our study. Thank you again for your valuable suggestions, which have significantly contributed to the improvement of our manuscript.

**Reviewer 2:**

1 There is no data on the functional status of the subjects .How many had poor functional status ? Dignity is proportional to independent status .Also gender of care giver ,whether care giver was a hired care giver ,spouse or daughter or son or daughter in law or a qualified nurse is important to know .Such a high prevalence of dermatitis has been reported .Were subjects using incontinence solutions such as diapers or leak proof pants ?

Response:Dear Reviewer,Thank you for your valuable comments. After thoroughly reflecting on and analyzing your suggestions, we provide the following response:Regarding the functional status, this study primarily focuses on the preliminary comprehensive analysis of dignity impairment symptoms in hospitalized elderly patients with incontinence-associated dermatitis. In our exclusion criteria, we have specifically excluded patients with severe physical disabilities, and this has been further explained in the study. Therefore, all participants have some level of self-care ability. It is important to note that all patients in this study are hospitalized, and the reasons for hospitalization are not necessarily directly related to incontinence-associated dermatitis. They may involve common conditions in elderly patients such as respiratory or neurological diseases. In the hospital environment, ethical principles must be adhered to, and all patients receive professional treatment and care, including medication and the use of incontinence care products. Additionally, we collected data on nursing frequency, severity of care, and other related information, which were analyzed for correlations. Therefore, this study does not involve changing treatment plans within the hospital setting.Regarding the primary caregiver’s identity, we mainly analyzed its potential impact on the patient’s psychological state from the perspective of social support. However, in the hospital environment, nearly all procedures are performed by healthcare professionals, as this is also governed by ethical principles.

After further analyzing your suggestions, we realized that your concerns may be more applicable to the elderly home care population. This is because once they leave the hospital treatment environment, they lack the timely guidance and treatment provided by the professional hospital team, and are more reliant on their self-care ability and family or social support. With your guidance, our team has gained new insights, and I believe that further research on home care elderly patients with incontinence-associated dermatitis is essential. Therefore, we plan to extend our study to home care elderly patients in the next phase, with the aim of exploring dignity impairment issues in different environmental contexts.Once again, thank you for your valuable guidance, which has played a crucial role in shaping our next steps for the study.

2 How was the cognitive status of elders who answered the dignity questionnaire ?were they literate or illiterate

Response:Dear Reviewer,Thank you for your valuable feedback and thoughtful consideration of our study. Regarding your concern about the cognitive status of elderly participants and the inclusion criteria, we would like to provide a detailed response.To ensure the accuracy of the multi-center survey data, a dedicated research team was established prior to the study initiation. Additionally, a survey coordinator was appointed at each hospital, and they underwent standardized training to ensure consistent data collection and quality control across all sites. The coordinators were responsible for ensuring that participants met the inclusion criteria and for maintaining data validity and reliability.According to our inclusion criteria, participants were required to be aged 60 or older, fully conscious, able to communicate effectively without cognitive impairment, and voluntarily provide informed consent. For illiterate participants, trained research assistants were available to provide verbal explanations and guidance to ensure they fully understood the questionnaire content and could respond accurately.We believe these measures were essential to ensure high-quality data collection, and we hope this clarifies your concern. Thank you once again for your insightful suggestions, which have helped to improve the rigor and quality of our study.

3 Very often dermatitis is due to candidal intertrigo which is curable with antifungal agents .Was there pain which impaired quality of life

Response:Dear Reviewer,Thank you for your insightful comment regarding the impact of pain on the quality of life in patients with incontinence-associated dermatitis (IAD). We appreciate your consideration of this important aspect of the condition.In our study, we did assess symptom distress as part of the dignity impairment evaluation, which reflects the discomfort, including pain, that patients may experience. However, symptom distress in our dignity assessment scale encompasses a broader range of discomfort, not limited to pain alone. While pain is certainly a significant factor in IAD-related distress, we did not specifically separate pain as an independent variable in our analysis. Instead, we included symptom distress as a comprehensive measure of discomfort, which indirectly captures the presence of pain associated with IAD.Based on your valuable feedback, we plan to give more attention to pain symptoms as an independent factor in our forthcoming longitudinal study. We will explore its specific impact on the quality of life in patients with IAD and refine our analysis to better address this crucial issue. We truly appreciate your guidance, which has helped shape the direction of our future research.

4 comparison of dignity questinnaire of incontinent people with dignity of a patient suffering from cancer is not appropriate

Response:Dear Reviewer,Thank you for your insightful comment regarding the comparison between dignity impairment in patients with incontinence-associated dermatitis (IAD) and those suffering from cancer. We appreciate your feedback on the appropriateness of such a comparison.We understand your concern, and we agree that it may not be entirely appropriate to compare dignity impairment in IAD patients directly with that of cancer patients, as these two groups have distinct clinical characteristics and disease trajectories. Our study specifically focused on elderly patients with IAD, and we initially drew comparisons with other patient populations, such as those suffering from cancer, to offer a broader context for understanding the dignity impairment rates in IAD.However, we recognize that a more relevant comparison might be with patients who have been treated in the ICU, particularly given the shared challenges of vulnerability, the need for extensive caregiving, and the higher sensitivity to issues like incontinence. ICU patients, who often experience critical illness and functional impairments, may face unique dignity-related challenges, including those arising from IAD caused by factors like enteral nutrition intolerance or diarrhea. This specific patient group would provide a more appropriate context for comparison, as both populations experience high levels of dependency and potential for dignity loss.We will revise the manuscript to focus on comparing dignity impairment in elderly patients with IAD and ICU patients, highlighting the shared challenges and unique factors that influence dignity in these two groups. All modifications will be marked in red font for clarity. We believe this approach will offer a clearer and more contextually relevant understanding of dignity impairment in elderly patients with IAD.Thank you again for your constructive feedback. We will ensure that the comparison is more appropriately framed in the revised manuscript.

**Reviewer 3:**

1 The research article exhibits several methodological and presentation-related errors that warrant attention. Firstly, the use of convenience sampling introduces selection bias, potentially limiting the generalizability of the findings. While the LASSO regression approach is appropriate, details on hyperparameter tuning and comprehensive multicollinearity diagnostics (e.g., variance inflation factor values) are missing, reducing the study's reproducibility.

Response:Dear Reviewer,Thank you for your valuable feedback. We acknowledge your concern regarding the use of convenience sampling and the potential for selection bias, which may limit the generalizability of our findings. Convenience sampling was employed due to the practical constraints of recruiting patients from multiple centers; however, we fully understand that this method may introduce certain biases. In response to your suggestion, we have added a detailed explanation of this limitation in the revised manuscript, specifically in the section discussing the study's limitations, highlighting the potential impact of this on the broader applicability of our results.Regarding the issue of multicollinearity diagnostics, we agree with your observation that this is a crucial aspect, particularly when using LASSO regression. While LASSO regression itself has inherent abilities to address collinearity, we recognize the importance of explicitly addressing multicollinearity in the analysis. Therefore, we have supplemented the Methods section to include details on how we assessed multicollinearity, including the use of Variance Inflation Factor (VIF) analysis. This additional explanation enhances the transparency and robustness of our statistical approach.We sincerely appreciate your thoughtful suggestions, which have contributed to improving the quality and rigor of our study.

2 The sample size slightly exceeds the calculated requirement without a clear explanation, and potential confounders, such as education level and urban/rural residence, are not fully addressed.

Response:Dear Reviewer,Thank you very much for your valuable comments on our study. First and foremost, we sincerely apologize for not adequately explaining the slight increase in the sample size beyond the calculated requirement. In this study, the primary reason for increasing the sample size was to ensure the stability and reliability of the research findings. By including a slightly larger sample, we were able to minimize the potential impact of random variations and missing data, thereby enhancing the robustness of our results. Based on your insightful suggestions, we have added a detailed explanation in the sample size calculation section of the manuscript, with all modifications highlighted in red font.

Regarding the potential confounders of education level and urban/rural residence, we did collect data on these factors during the study. The general data analysis indicated no statistically significant difference in education level. Although differences in urban and rural residence showed statistical significance in univariate analysis, this factor did not exhibit a statistically significant effect on dignity impairment symptoms in elderly patients with incontinence-associated dermatitis in the multivariate analysis, and therefore did not impact the conclusions of the study.We deeply appreciate your comments, which are crucial for enhancing the quality and rigor of our paper. Once again, thank you for your meticulous review and valuable suggestions. We sincerely hope this response sufficiently addresses your concerns.

3 The discussion lacks depth in contextualizing findings, particularly in offering actionable interventions for identified protective and risk factors. Ethical considerations, while mentioned, do not detail measures taken to mitigate psychological distress during data collection.

Response:Dear Reviewer,Thank you for your valuable comments regarding the depth of our discussion and the ethical considerations in our study. In response, we have revised the discussion section to incorporate a more in-depth analysis of the findings, focusing on actionable interventions for the identified protective and risk factors, as guided by your suggestions. Additionally, we have supplemented the ethical statement with details on the measures taken during the research process, including the anonymous processing of all participant information and steps to ensure privacy and voluntary participation. All modifications have been marked in red within the manuscript for clarity. We sincerely appreciate your constructive feedback, which has significantly enhanced the quality and rigor of our manuscript.

4 Additionally, vague statements about data availability ("on reasonable request") hinder transparency, and referenced figures and tables are absent in the manuscript, impacting the clarity of the presented results. Minor grammatical inconsistencies and overly complex sentences further detract from the article's readability and coherence.

Response:Dear Reviewer,Thank you for your valuable comments on this study. In response to your feedback, we have made the following revisions. Firstly, regarding data availability, we have amended the statement to "Supporting data for this study are available from the corresponding author upon request." to enhance transparency and reproducibility. Secondly, regarding the issue with the figures and tables, due to the submission requirements of the journal, we uploaded all figures and tables separately, which resulted in their absence in the manuscript. We sincerely apologize for this inconvenience. All figures and tables have been uploaded as separate attachments. Finally, with regard to grammar and expression issues, we have thoroughly reviewed the manuscript to correct any grammatical errors and simplified some sentences to make the content more concise and clearer.We appreciate your meticulous review of our study. Your suggestions have played an important role in improving and refining the manuscript.

**Reviewer 4:**

1 It is not prudent to specify a statistical test in the title, even if it may be informative.

Response:Dear Reviewer,In response to your suggestion, and based on the recommendations from the reviewers and editor, we have revised the title of the manuscript to "Factors Influencing Dignity Impairment in Elderly Patients with Incontinence-Associated Dermatitis: A Lasso and Logistic Regression Approach." We understand your concern regarding the inclusion of specific statistical methods in the title. However, we believe that this revision helps clearly convey the advanced analytical techniques employed in the study while addressing the focus of the research. We appreciate your input, which has contributed to enhancing the clarity of the manuscript.Thank you once again for your thoughtful comments.

2 In the introduction, the importance of the study is not adequately specified. It is important to expand the topic and references based on points such as the management of dermatological diseases in older adults, the quality of life in people with urinary incontinence.

Response:Dear Reviewer,Thank you very much for your valuable feedback. We fully agree with your suggestion regarding the inadequacy of specifying the importance of the study in the introduction. In response, we have expanded the discussion, particularly focusing on the management of dermatological diseases in older adults and the quality of life in people with urinary incontinence. We have highlighted the impact of these issues on the overall health and quality of life of elderly patients and have added relevant references to support these points. This enhancement helps to more comprehensively present the background and significance of our study, emphasizing its clinical relevance and practical importance for the elderly population. All modifications have been marked in red for your convenience. We sincerely appreciate your guidance, which has greatly contributed to improving the quality and rigor of our manuscript.

3 The study design is not defined.

Response:Dear Reviewer,Thank you for your valuable feedback on our study. We sincerely apologize for the lack of clarity in the research design, as pointed out in your comments. Following your guidance, we have made appropriate revisions and additions to the "Methods" section of the abstract. Additionally, we have further clarified the study design, sample selection process, and the use of survey tools. All the changes have been marked in red font for your easy reference.We greatly appreciate your thorough review and insightful suggestions, which have significantly contributed to enhancing the scientific rigor and accuracy of our paper.

4 It is understood that if the study is analytical according to the title, a sample size for only one population should not be used.

Response:Dear Reviewer,Thank you for your valuable feedback. We sincerely apologize for the concern you raised regarding the sample size for a single population in an analytical study. In the design of our research, we focused on elderly patients with incontinence-associated dermatitis, specifically examining the symptoms of dignity impairment within this group. The sample size was calculated to meet the statistical analysis requirements for this specific population. We understand that when conducting analytical studies, it is crucial to consider the sample sizes across different populations.In response to your insightful suggestion, we have added clarification in the introduction of the paper, where we emphasize that the analysis of this study focuses on a single population. Additionally, we provide the rationale for selecting this group and its significance in the context of the research. We recognize that your feedback has significantly contributed to enhancing the scientific rigor of the paper, and your suggestions have provided valuable direction.All modifications have been highlighted in red font. Once again, thank you for your thorough review and valuable advice. Your guidance has been instrumental in improving the quality of our paper.

5 In the exclusion criteria, it is not specified what types of incurable or severe diseases, the presence or absence of disability or functional impairment, and whether they had any other type of dermatological condition.

Response:Dear Reviewer,Thank you for your valuable feedback. We sincerely apologize for not specifying the types of incurable or severe diseases, the presence or absence of disability or functional impairment, and whether other dermatological conditions were considered in the exclusion criteria. In response to your suggestion, we have revised and expanded the exclusion criteria to provide clearer details regarding these aspects.In the revised exclusion criteria, we have specified the types of incurable or severe diseases, clarified the definition of disability or functional impairment, and explicitly excluded participants with other dermatological conditions. This ensures the specificity and consistency of the study population.All modifications have been highlighted in red font. Once again, thank you for your meticulous review and valuable suggestions. Your guidance has greatly helped us improve the accuracy and completeness of the paper.

6 It is not specified when talking about the dignity test the cutoff point for evaluating its deterioration.

Response:Dear Reviewer,Thank you for your valuable feedback. Regarding the point you raised about the lack of a specified cutoff point for evaluating the deterioration of dignity in the dignity test, we apologize for the oversight. In response to your suggestion, we have provided a detailed explanation in the manuscript, clarifying the cutoff point used in the dignity assessment tool. Additionally, we have included supporting references to justify the selection of this cutoff point and its application in similar studies.All modifications are marked in red font. Thank you once again for your thorough review and invaluable advice, which has greatly contributed to improving the scientific quality and accuracy of our paper.

7 In the statistical section, they detail the type of analysis and the procedure for a multivariate analysis. It is also important to detail the assumptions for conducting this analysis and whether all of them were met.

Response:Dear Reviewer,Thank you for your valuable feedback. Regarding your point about the lack of detail on the assumptions for conducting multivariate analysis, we have carefully considered your suggestion and made the necessary additions. In the revised statistical section, we have provided a detailed explanation of the variable selection process and the collinearity diagnostics performed prior to conducting binary logistic regression. We used variance inflation factors (VIF) to assess collinearity, and any variables with a VIF greater than 10 were excluded to avoid multicollinearity. Additionally, we included a description of how the Hosmer-Lemeshow goodness-of-fit test was used to assess model fit and explained the criteria for evaluating the model's performance based on the test results.All modifications are marked in red font. Thank you once again for your thorough review and invaluable suggestions, which have greatly enhanced the scientific rigor and accuracy of our study.

8 Urinary incontinence is not evaluated as a confounding variable because it can itself alter the patient's dignity, as the test assesses dignity in a global manner and not defined by diseases.

Response:Dear Reviewer,Thank you for your valuable comment. Regarding whether urinary incontinence should be considered a confounding variable, we understand your concern. We did not evaluate urinary incontinence as a confounding variable in this study for the following reasons:Firstly, the aim of this study was to explore various factors associated with dignity impairment. Dignity, as a comprehensive and global concept, encompasses multiple dimensions (such as psychological status, social support, symptom distress, etc.). The Dignity Inventory tool, used for measuring dignity, is a comprehensive assessment tool designed to evaluate an individual's dignity from various aspects. Therefore, we consider urinary incontinence itself as part of dignity impairment. It significantly impacts dignity within the dimensions of dignity impairment and should not be regarded as an independent confounding variable. In other words, urinary incontinence is one of the primary factors under investigation in this study concerning its effect on patients' dignity.Secondly, while urinary incontinence is an important factor affecting the quality of life of elderly patients, this study focuses on a comprehensive evaluation of dignity impairment symptoms, rather than being limited to any specific disease or symptom. Since urinary incontinence is a core factor within the dignity impairment assessment, we believe that not treating it as a confounding variable in statistical analysis does not undermine the scientific integrity of the study.We understand your concerns and believe that this issue offers valuable insight for further improving our research. Therefore, based on your constructive suggestion, we plan to conduct a more in-depth analysis of this factor in our future longitudinal research exploring causal relationships.Thank you once again for your thorough review and valuable suggestions. Your guidance has been instrumental in enhancing the quality of the paper.

9 In the discussion: the comparison should ideally be with chronic and/or dermatological pathologies in this population.

Response:Dear Reviewer,Thank you for your valuable feedback. Regarding your suggestion to compare our findings with chronic and/or dermatological pathologies, we have made revisions to address this. In the discussion section, we chose to compare our study group with critically ill patients. This decision was made based on the fact that many critically ill patients require enteral nutrition and are unable to care for themselves, often experiencing gut microbiome dysbiosis and other factors that are associated with incontinence-associated dermatitis. By making this comparison, we aimed to explore the impact of different disease states on dignity impairment symptoms and highlight the significance of this comparison in the context of our study.All modifications have been marked in red font. Thank you once again for your thorough review and invaluable suggestions, which have greatly enhanced the quality of our paper.

10 Explain how they addressed the limitations and strengths of the study.

Response:Dear Reviewer,Thank you for your valuable comment. Regarding the explanation of the limitations and strengths of the study, we have addressed these aspects in the manuscript. In terms of limitations, we have mentioned potential factors that may affect the interpretation of the results and suggested that future research could improve the design and methodology to overcome these limitations. Regarding the strengths of the study, we highlighted several important contributions. First, this study addresses the psychological health issues of elderly patients with incontinence-associated dermatitis at an early stage, filling a gap in this field and providing valuable insights for clinical healthcare providers in making medical decisions. Second, the study utilized a multi-center, large sample design, along with comprehensive assessment tools and rigorous statistical methods, all of which enhanced the scientific rigor and reliability of the findings. Thank you again for your attention to this section. We will continue to work on further improving the study.

We tried our best to improve the manuscript and made some changes in the manuscript. These changes will not influence the content and framework of the paper.

We appreciate for Editors/Reviewers’ warm work earnestly, and hope that the correction will meet with approval.

Once again, thank you very much for your comments and suggestions.

Thank you and best regards.

Jinlei Du
